# Supplementary material for: Identification of photoperiod-induced specific miRNAs in the adrenal glands of Sunite sheep (Ovis aries)
Source: Front Vet Sci. 2022 Jul 22;9:888207. doi: 10.3389/fvets.2022.888207 (PMC9354845; doi:10.3389/fvets.2022.888207)
Supplement: Supplementary file 1 [file Data_Sheet_1.ZIP › Supplementary file/miRDeep2 file.zip!/miRDeep2 file/Know_Structure/LP42A2/oar-mir-1197.pdf]

miRBase precursor : oar-mir-1197
Total read count : 71
oar-miR-1197-5p read count : 0
oar-miR-1197-3p read count : 59
remaining reads : 12

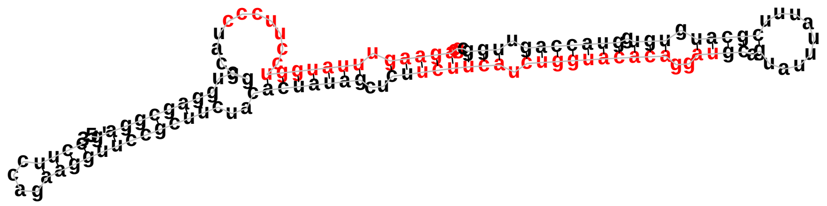

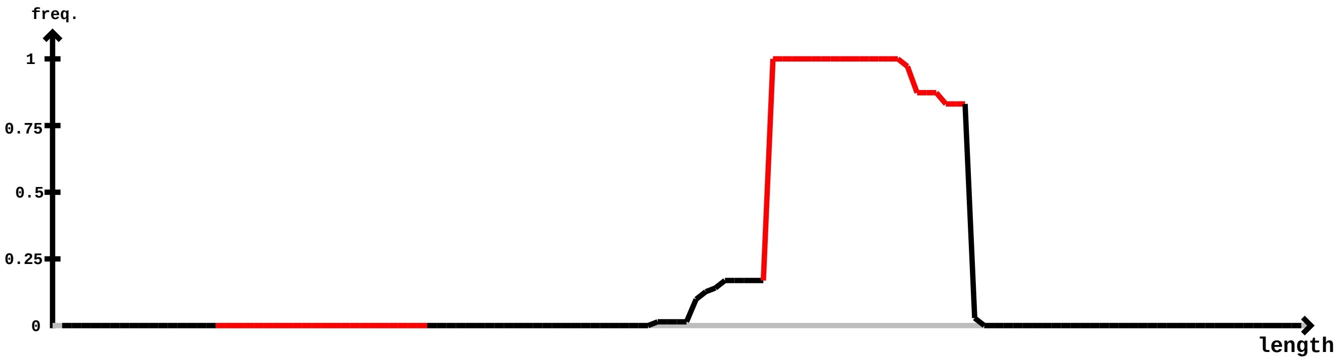

oar-miR-1197-3p

oar-miR-1197-5p

```
5'- gaggcgaggucggcaucccuuccugguauuugaagacgcgguugaccauggugugua cgcuuuauuuaugacguaggacacauggucuacuucuucucgauaucacaucuucgccuuggaagaccuucc  -3'   exp
    (((((((((..(...........(((((((.(((((...(((.(((((((.(((.(((((.........).))))..)))))))))).))))))))..))))))))..)))))))))((((....))))   reads   mm
    .............................................................uuauuuaugacguaggacacauggucu.........................................   1       0
    .................................................................uuaugacguaggacacaugguc..........................................   2       0
    .................................................................uuaugacguaggacacauggucu.........................................   4       0
    ..................................................................uaugacguaggacacauggucu.........................................   2       0
    ...................................................................augacguaggacacauggucuacu......................................   1       0
    ....................................................................ugacguaggacacauggucuacu......................................   2       0
    .........................................................................uaggacacauggucuacuucu...................................   57      0
    .........................................................................uaggacacauggucuacuucuu..................................   2       0
```
